# Late arrhythmic burden in patients with left bundle branch block after TAVR with the Evolut valve

**DOI:** 10.1093/europace/euaf057

**Published:** 2025-03-19

**Authors:** Silvia Mas-Peiro, Thibault Lhermusier, Marina Urena, Luis Nombela-Franco, Victoria Vilalta, Antonio Muñoz-Garcia, Ignacio Amat-Santos, Felipe Atienza, Neal Kleiman, Chekrallah Chamandi, Vicenç Serra, Marc W Deyell, Francisco Campelo-Parada, Pierre Mondoly, Gaspard Suc, Victoria Canadas-Godoy, Eduard Fernandez-Nofrerias, Javier Castrodeza, Jaime Elizaga, Pierre Baudinaud, Jaume Francisco Pascual, John G Webb, Emilie Pelletier-Beaumont, François Philippon, Josep Rodés-Cabau

**Affiliations:** Department of Cardiology, Quebec Heart & Lung Institute, Laval University, 2725 Chemin Ste-Foy, Quebec City, Quebec, Canada G1V4G5; Department of Cardiology, Hôpital Universitaire de Toulouse, Toulouse, France; Department of Cardiology, Assistance Publique-Hôpitaux de Paris, Hôpital Bichat-Claude Bernard, Paris, France; Instituto Cardiovascular, Hospital Clinico San Carlos, IdISSC, Madrid, Spain; Department of Cardiology, Hospital Germans Trias i Pujol, Badalona, Spain; Department of Cardiology, Hospital Virgen de la Victoria, Málaga, Spain; Department of Cardiology, Hospital Universitario de Valladolid, Valladolid, Spain; Department of Cardiology, Hospital Gregorio Marañón, Madrid, Spain; Department of Cardiology, Houston Methodist DeBakey Heart and Vascular Center, Houston, TX, USA; Department of Cardiology, Hôpital Européen George Pompidou, Paris, France; Department of Cardiology, Hospital Universitari Vall d’Hebron, CIBER-CV, Barcelona, Spain; Department of Cardiology, St Paul’s Hospital, Vancouver, Canada; Department of Cardiology, Hôpital Universitaire de Toulouse, Toulouse, France; Department of Cardiology, Hôpital Universitaire de Toulouse, Toulouse, France; Department of Cardiology, Assistance Publique-Hôpitaux de Paris, Hôpital Bichat-Claude Bernard, Paris, France; Instituto Cardiovascular, Hospital Clinico San Carlos, IdISSC, Madrid, Spain; Department of Cardiology, Hospital Germans Trias i Pujol, Badalona, Spain; Department of Cardiology, Hospital Universitario de Valladolid, Valladolid, Spain; Department of Cardiology, Hospital Gregorio Marañón, Madrid, Spain; Department of Cardiology, Hôpital Européen George Pompidou, Paris, France; Department of Cardiology, Hospital Universitari Vall d’Hebron, CIBER-CV, Barcelona, Spain; Department of Cardiology, St Paul’s Hospital, Vancouver, Canada; Department of Cardiology, Quebec Heart & Lung Institute, Laval University, 2725 Chemin Ste-Foy, Quebec City, Quebec, Canada G1V4G5; Department of Cardiology, Quebec Heart & Lung Institute, Laval University, 2725 Chemin Ste-Foy, Quebec City, Quebec, Canada G1V4G5; Department of Cardiology, Quebec Heart & Lung Institute, Laval University, 2725 Chemin Ste-Foy, Quebec City, Quebec, Canada G1V4G5

**Keywords:** Conduction disturbances, Evolut R, Left bundle branch block, Pacemaker implantation, Transcatheter aortic valve replacement

## Abstract

**Aims:**

Arrhythmic burden after discharge in patients with new-onset persistent left bundle branch block (NOP-LBBB) following transcatheter aortic valve replacement (TAVR) with Evolut devices remains largely unknown. The aim of this study is to assess the incidence and type of arrhythmias at 2-year follow-up in patients with NOP-LBBB post-TAVR.

**Methods and results:**

This is a prospective multicentre study including 88 patients with LBBB persisting for ≥3 days post-implantation. Before discharge, an implantable loop recorder (REVEAL XT/LINQ) was implanted; patients had continuous monitoring for 2 years. Arrhythmic events were adjudicated in a central core lab. Of the arrhythmic events, 411 were detected in 58 patients [65.9%; 2 (1–4) events per patient]. Symptoms were reported in 12/58 (20.7%), and therapy was changed in 25/58 (43.1%). There were 101 bradyarrhythmic events in 33 patients [35 high-grade atrioventricular block (HAVB) and 66 severe bradycardia]. The HAVB incidence was higher in the early (4-week) phase and remained stable over time, whereas severe bradycardia increased after 1 year. Permanent pacemaker was required in 11 (12.5%) patients (6.8% and 5.7% in the first and second year, respectively). There were 310 tachyarrhythmic events in 29 patients (120 AF/AFL, 111 AT, 72 SVT, 6 NSVT, and 1 VT); its incidence decreased throughout the 2 years. New AF/AFL episodes occurred in 20/69 patients [29%; symptomatic in 2/20 (10%)].

**Conclusion:**

Patients with NOP-LBBB post-TAVR with Evolut devices exhibited a high burden of late arrhythmias, with events occurring in two-thirds of patients and leading to treatment changes in about half of them. These data should inform future studies on cardiac monitoring devices for follow-up and treatment optimization in this challenging population.

## Graphical Abstract

**Graphical Abstract:**
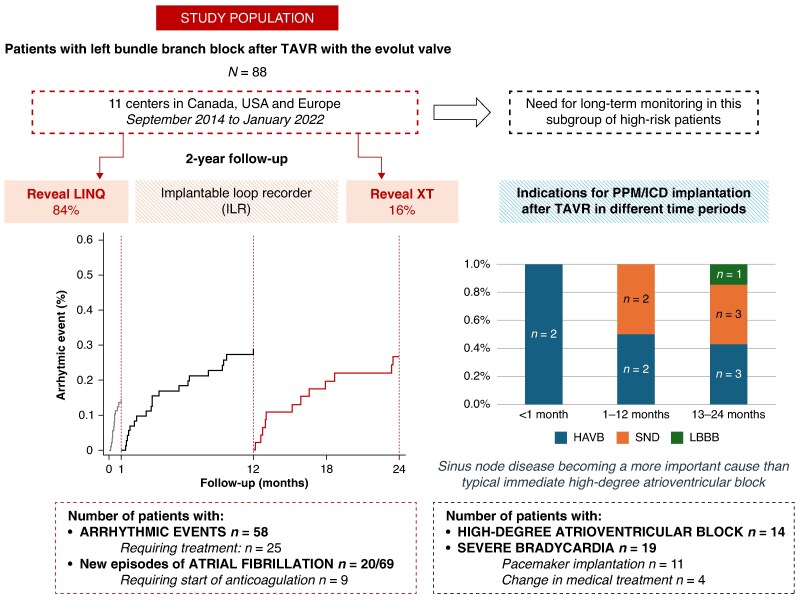

**What’s new?**

- Late-appearing arrhythmic events are particularly common in the long term in patients showing new-onset persistent LBBB after TAVR with the Evolut system.
- Treatment changes due to arrhythmia are still needed at long-term follow-up in about half of the patients.
- Late need for PPM occurs frequently at long-term follow-up after TAVR, with sinus node disease becoming a more important cause than typical immediate high-degree atrioventricular block.
- New AF/AFL episodes are frequent and usually require the onset of an anticoagulation regimen.
- Ventricular arrhythmias are rare in the long-term post-TAVR.

## Introduction

The occurrence of conduction disturbances, particularly left bundle branch block, remains the most common complication of transcatheter aortic valve replacement (TAVR),^1^ and it has been associated with an increased risk of mortality and heart failure hospitalization at follow-up.^2,3^ Thus, timely identification and appropriate management are critical in this high-risk group of patients. The use of some self-expanding valves such as the CoreValve system (Medtronic, Minneapolis, MN) has been classically associated with an increased risk of conduction disturbances compared to balloon-expandable valves.^4,5^ Major changes in the stent frame have been implemented to the newer generation CoreValve Evolut system, and controversial data have been reported regarding the changes in the incidence of conduction disturbances with the use of this new valve platform.^6–10^ However, in those patients with new-onset persistent left bundle branch block (NOP-LBBB), data on the occurrence of arrhythmias (including life-threatening events) detected by continuous electrocardiogram (ECG) monitoring beyond the in-hospital period have been scarce and limited to a relatively short (1 month) follow-up period.^11,12^

The use of implantable cardiac monitoring devices has been shown to be useful for detecting cardiac arrhythmias in different clinical scenarios,^13^ but data in the TAVR field have been restricted to studies including a mix of older and newer valve generations and different valve types.^14^ Thus, the aim of this study was to determine both the incidence and type of arrhythmias at 2-year follow-up with the use of continuous ECG monitoring in patients with NOP-LBBB following TAVR with the Evolut valve system.

## Methods

This was a prospective multicentre study including consecutive patients who underwent TAVR with the Evolut valve system between September 2014 and January 2022 in 11 centres in Canada, the USA, and Europe and had NOP-LBBB that persisted ≥3 days following the procedure. Patients receiving other types of valve prostheses were excluded from the analysis. An implantable loop recorder (ILR), either a Reveal ICM XT or a LINQ (Medtronic) device, was implanted before discharge in all patients. Left bundle branch block (LBBB) was defined according to the American College of Cardiology/American Heart Association/Heart Rhythm Society (ACC/AHA/HRS) recommendations.^15^ A 2-year follow-up was performed in all patients with outpatient visits and 12-lead ECG being recorded at 1, 12, and 24 months. Data from patients who received the Reveal LINQ device were obtained through automatic wireless transmission, whereas data from patients with a Reveal XT device were generated by device interrogation every 3 months. To address the scheduling constraint of the Reveal XT device, the systematic recording of atrial fibrillation events was deactivated by the centres for patients known to have chronic atrial fibrillation in order to avoid missing the recording of other new events. No adjustment was required for the Reveal LINQ device, since events are transmitted on a daily basis. Valve Academic Research Consortium 2 (VARC-2) criteria were used to define clinical events.^16^

The primary endpoint of the study was the incidence of arrhythmic events leading to a treatment change at 2-year follow-up. Secondary endpoints included significant arrhythmias defined according to the ACC/AHA/HRS guidelines.^17^ These were classified as (i) significant bradyarrhythmia [high-degree atrioventricular block (HAVB), severe bradycardia (heart rate < 30 bpm in four consecutive beats or pause > 3 s)]; (ii) atrial fibrillation (AF)/atrial flutter (AFL)/atrial tachycardia (AT)/supraventricular tachycardia (SVT) episodes lasting >30 s; (iii) ventricular tachycardia (VT) (non-sustained: lasting between 6 and 30 s; sustained: lasting >30 s); and (iv) ventricular fibrillation (VF). More specifically, high-degree AVB was defined as the presence of ≥2 consecutive, non-conducted P waves (high-grade second degree) and as unrelated P waves to the QRS complexes (third degree). Arrhythmic burden adjudication was performed in a central core lab. Indications for PPM implantation were also based on ACC/AHA/HRS recommendations. Class I, IIa, and IIb recommendations were followed by managing physicians at each participating centre for sinus node dysfunction, acquired atrioventricular block, and chronic bifascicular block.

The initial diagnosis and management of arrhythmic events were the responsibility of the investigators of each participating centre, based on the mentioned guidelines and on current clinical practice.

The study was approved by the institutional ethics committee of participating centres, and all patients provided signed informed consent. The results including the arrhythmic burden at 1- and 2-year follow-up of 35 patients included in the present study had been previously reported.^18^

### Statistical analysis

Numbers and frequencies were used to report categorical variables. Continuous variables were presented as mean ± standard deviation when normally distributed and as median (interquartile range) if non-normally distributed. *χ*² tests (or Fisher’s tests when appropriate) were used to compare categorical variables. Numerical variables were compared using the Student’s *t*-test or Wilcoxon test as appropriate. Kaplan–Meier curves were plotted for event rates over time. Statistical significance was set at *P* < 0.05. All analyses were performed with the SPSS statistical software package version 29.0.

## Results

A total of 88 Evolut recipients (Evolut R: 87, Evolut PRO: 1) with NOP-LBBB after TAVR were included. The main baseline characteristics and procedural data are shown in *Table 1*. Median (IQR) age of the study population was 83 (80–86) years; 72.7% were women; and the median (IQR) STS-PROM score was 4.6 (3.3–7.0).

**Table 1:** Baseline and procedural characteristics of the population

	Overall cohort (*n* = 88)
Age (years)	83 (80–86)
Female, *n* (%)	64 (72.7)
Diabetes mellitus	30 (34.1)
Atrial fibrillation/flutter, *n* (%)	19 (21.6)
Paroxysmal	8 (42.1)
Permanent	11 (57.9)
STS-PROM score (%)	4.6 (3.3–7.0)
CHADS-VASc2 score (%)	4.5 (4.0–5.0)
ECG	
PR interval (ms)	167 (153–192)
QRS duration (ms)	94 (80.0–100.0)
Echocardiography	
LVEF (%)	60 (55–61.2)
Mean gradient (mmHg)	40.5 (30.9–48)
Aortic valve area (cm^2^)	0.71 (0.54–0.87)
Approach	
Transfemoral	84 (95.5)
Transapical/transaortic	1 (1.1)
Subclavian/transcarotid	3 (3.4)
New-onset persistent LBBB	
PR interval (ms)	194 ± 46
QRS duration (ms)	140 (130–156)
Time to implantable monitor, days post-TAVR	4.8 (3–6)
Type of device, LINQ/XT	84%/16%
Hospitalization length (days)	6 (4–7)

Values are shown as *n* (%) for qualitative variables; mean ± SD for normally distributed quantitative variables; or median (interquartile range) for non-normally distributed quantitative variables.

ECG, electrocardiogram; LBBB, left bundle branch block; LVEF, left ventricular ejection fraction; STS-PROM, Society of Thoracic Surgeons Predicted Risk of Mortality.

### Global arrhythmic burden

Overall, 411 new arrhythmic events were detected in 58 patients (65.9%) within the total 2-year post-TAVR follow-up, with a median number of 2 (1–4) events per patient. Symptoms were reported in 12 out of 58 patients (20.7%) and a change in treatment was required in 25 out of 58 patients (43.1%).

The following types of arrhythmic events were found: 310 tachyarrhythmic events including 120 AF/AFL, 111 AT, 72 SVT, 6 non-sustained ventricular tachycardia (NSVT), and 1 VT and 101 bradyarrhythmic events in 33 patients, including 35 HAVB events and 66 severe bradycardia events. Complete details of arrhythmic events can be found in *Table 2* and Supplementary material online, *Table S1*. Duration and symptoms of all severe bradycardia, non-sustained ventricular tachycardia, and atrial fibrillation/atrial flutter events are shown in Supplementary material online, *Table S2*.

**Table 2:** Number of patients with arrhythmic events in early (≤1 year) and late follow-up (>1 year) (*n* = 88)

	Overall period	First year	Second year
Global arrhythmic burden			
Patients with arrhythmic events	58	33	25
Arrhythmic events per patient	2 (1–4)	2 (1–4)	3 (1–4)
Patients with arrhythmic events requiring treatment	25	16	9
Bradyarrhythmias			
Patients with bradyarrhythmic events	33	19	17
Patients with high-degree atrioventricular block	14	8	6
Patients with severe bradycardia	19	11	11
Patients with bradyarrhythmias requiring treatment	15	10	5
Pacemaker implantation^a^	11	6	5
Change in medical treatment	4	4	0
Tachyarrhythmias			
Atrial fibrillation/atrial flutter			
Patients with new episodes of atrial fibrillation/atrial flutter^b^	20/69	13/69	7/69
Patients with new episodes of atrial fibrillation/atrial flutter leading to anticoagulation therapy	9	5	4
Ventricular tachycardia			
Patients with episodes of ventricular tachycardia	6	4	2
Ventricular tachycardia episodes per patient	1 (1–1)	1 (1–1)	1 (1–1)
Patients with ventricular tachycardia episodes who had a treatment modification	2	2	0
Cardiac resynchronization therapy-defibrillator (CRT-D)^c^	2	0	2

Values are expressed as *n*, *n*/*n*, or median (interquartile range).

^a^All pacemakers were implanted with an RV apical lead; no leadless or conduction system pacing was used.

^b^Only patients without prior atrial fibrillation are considered in the denominator for the percentage.

^c^CRT-D was implanted based on heart failure, not on ventricular arrhythmias.

**Figure 1:**
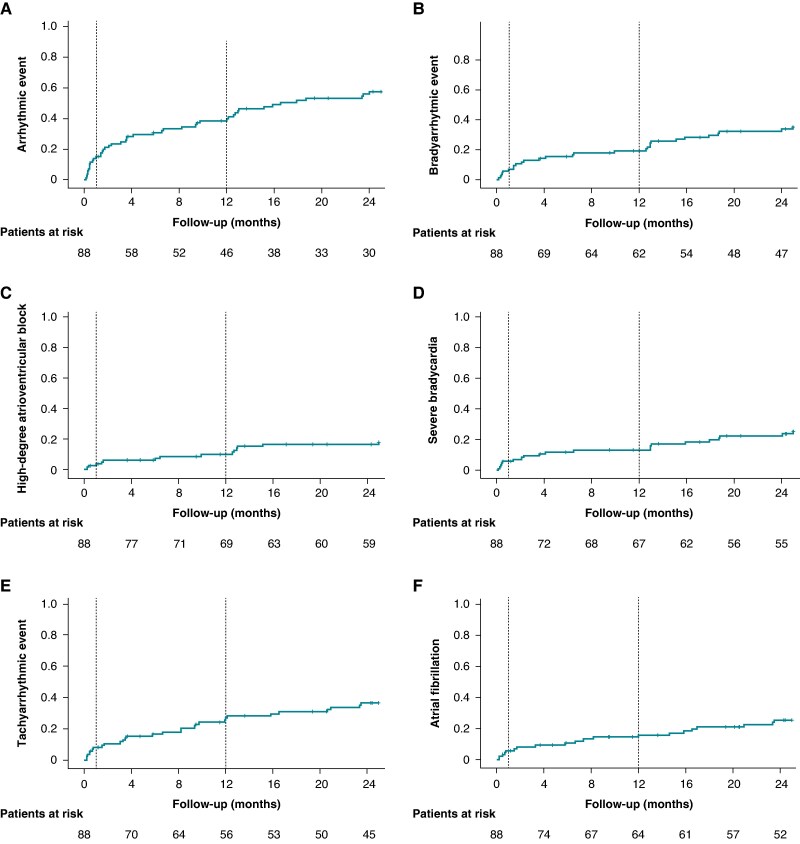
Time to first arrhythmic episode in patients receiving Evolut system devices. (*A*) Time to the first arrhythmic (brady- or tachyarrhythmia) episode. (*B*) Time to the first episode of bradyarrhythmia. (*C*) Time to the first episode of high-degree atrioventricular block. (*D*) Time to the first episode of severe bradycardia. (*E*) Time to the first episode of tachyarrhythmia. (*F*) Time to the first episode of atrial fibrillation/atrial flutter.

The time to first arrhythmic event is shown in Kaplan–Meier curves in *Figure 1*. Of the 411 arrhythmic events, 104 (25.3%) were detected in 25 out of 58 patients (43.1%) after the first year. Further details regarding distribution of events within the first or second year of follow-up are shown in Supplementary material online, *Table S3*.

### Bradyarrhythmic events and need for pacemaker implantation

All bradyarrhythmic events occurring within the 2-year period are reported in Supplementary material online, *Table S1*. A bradyarrhythmic event was seen in 33 patients, but only 9 were symptomatic. The proportion of patients with severe bradycardia was higher than the proportion of patients with HAVB both in first and second years. Time to first bradyarrhythmic episode is shown in *Figure 1B*. Details on patients developing complete AVB can be found in Supplementary material online, *Table S4*. Bradyarrhythmic events showed different occurrence patterns in the first and second year, with a stable trend in HAVB events and an increasing trend for severe bradycardia in the second year of follow-up.

During the first year, bradyarrhythmic events occurred in 19 patients, with 10 patients requiring a change in treatment and only 3 being symptomatic. After the first year, bradyarrhythmic events occurred in 17 patients, with 5 patients requiring treatment. The PPM implantation in order to treat bradyarrhythmia was needed in six patients within the first year and in five patients during the second year. Only four of them showed symptoms. All patients received a standard right ventricular (RV) apical pacing. There were no cases of leadless or conduction system pacing. It is worth mentioning that not all patients with severe bradycardia underwent pacemaker implantation.

A cardiac resynchronization therapy defibrillator (CRT-D) was needed in two patients. One of them had a baseline ventricular dysfunction due to known ischaemic cardiomyopathy; the other one showed progressive ventricular dysfunction during follow-up after TAVR. While PPM implantation was distributed similarly between Year 1 and Year 2, all CRT-D implantations occurred within the second year of follow-up, due to a systolic dysfunction caused by ventricular dyssynchrony secondary to LBBB.

The reasons for PPM/CRT-D implantation were mainly HAVB or sinus node disease (SND). The PPM implantations within the first month were all due to HAVB, whereas indications in patients who required a PPM at a later stage were mainly due to either HAVB or SND in similar proportions. Of note, LBBB resolved only temporary in one patient and permanently in two patients receiving a PPM. Further details on post-procedural and follow-up ECGs in patients requiring a PPM or a CRT-D are shown in *Table 3*.

**Table 3:** Electrocardiograms post-procedure, at 30 days, 12 months, and 24 months for all patients requiring a permanent pacemaker or a cardiac resynchronization therapy defibrillator

Pat.	Timing	Days after TAVR	Pacemaker indication	Symptoms	Heart rate	Heart rhythm	PR duration	AVB	QRS duration	QRS morphology	LBBB resolution	QRS axis
**1**	Post-procedure	220	Prolonged ventricular pauses									
	Discharge				85	AF		None	132	LBBB	No	Normal
	30 days				81	AF			127	LBBB	No	Normal
	12 months				94	AF			138	LBBB	No	Normal
	24 months				66	AF			136	LBBB	No	Normal
**2** ^a^	Post-procedure	393	HAVB and left ventricular dysfunction									
	Discharge				100			None		LBBB	No	Left
	30 days											
	12 months					Sinusal				LBBB		
	24 months											
**3**	Post-procedure	48	Slow atrial fibrillation									
	Discharge					AF		None	134	LBBB	No	Left
	30 days				93	AF		None	120	ILBBB	No	Left
	12 months				96	AF		None	86			Left
	24 months				80	AF						
**4**	Post-procedure	7	CHB	Syncope								
	Discharge				73	Sinusal	202	1st degree	146	LBBB	No	Left
	30 days				66	Sinusal	188	None	146	LBBB	No	Left
	12 months				64	Sinusal	214	1st degree	142	LBBB	No	Normal
	24 months											
**5** ^a^	Post-procedure	596	LBBB and left ventricular dysfunction									
	Discharge				78	Sinusal	224	1st degree	178	LBBB	No	Left
	30 days				91	Sinusal	204	1st degree	164	LBBB	No	Left
	12 months				75	Sinusal	220	1st degree	174	LBBB	No	Left
	24 months				84	Paced	164		148			
**6**	Post-procedure	41	CHB	Lipothymia/syncope								
	Discharge				75	Sinusal	240	1st degree	124	LBBB	No	Left
	30 days				65	Sinusal	232		116			
	12 months											
	24 months				87	Paced	192		120			
**7**	Post-procedure	9	AVB	Syncope								
	Discharge				101	Sinusal	202	1st degree	130	LBBB	No	
	30 days											
	12 months											
	24 months											
**8**	Post-procedure	571	Sinus dysfunction, significant pauses									
	Discharge				57	Sinusal						
	30 days				75	Sinusal	173	None	154	LBBB	No	Undetermined
	12 months				60	Sinusal	174	None	157	LBBB	No	Left
	24 months				55	Paced	202	1st degree	161	Paced	No	Normal
**9**	Post-procedure	483	Pause > 6 s									
	Discharge				55	Sinusal						
	30 days				53	Sinusal	183		158	LBBB	No	
	12 months				75	Sinusal		None		LBBB	No	Normal
	24 months				81	AF		None	144	LBBB	Yes	Normal
**10**	Post-procedure	633	Pause > 20 s	Pre-syncope								
	Discharge				55	AF		None	180	LBBB	No	Normal
	30 days				60	AF		None	100	LBBB	Yes	Normal
	12 months				60	AF		None	100	ILBBB	Yes	Normal
	24 months				70	AF			110	ILBBB	Yes	Normal
**11**	Post-procedure	195	HAVB									
	Discharge				51	AF	250	1st degree	152	LBBB	No	Normal
	30 days											
	12 months											
	24 months				72	Paced				Paced		Left
**12**	Post-procedure	461	CHB									
	Discharge				75	Sinusal	250	1st degree		LBBB	No	Undetermined
	30 days				72	Sinusal	210	1st degree	110	IVCD	Yes	Normal
	12 months				67	Sinusal	228	1st degree	166	LBBB	No	Left
	24 months				60	Sinusal	200	1st degree	162	LBBB	No	Normal
**13**	Post-procedure	399	HAVB									
	Discharge				81	Sinusal	188		140	LBBB	No	Left
	30 days				83	Sinusal	158		144	LBBB	No	Left
	12 months				73	Sinusal	162		146	LBBB	No	Left
	24 months				72	Sinusal	188		132	LBBB	No	Left

Some ECGs were not available due to the COVID pandemic.

AF, atrial fibrillation; CHB, complete heart block; CRT-D, cardiac resynchronization therapy-defibrillator; ECG, electrocardiogram; HAVB, high-degree atrioventricular bloc; ILBBB, incomplete left bundle branch block; IVCD, intraventricular conduction delay; LBBB, left bundle branch block; PPM, pacemaker implantation; TAVR, transcatheter aortic valve replacement.

^a^Patients with CRT-D.

### Tachyarrhythmic events

A total of 29 patients showed a tachyarrhythmic event. All tachyarrhythmic events occurring within the 2-year period are reported in Supplementary material online, *Table S1*. Time to first tachyarrhythmic episode is shown in *Figure 1E*. New episodes of AF/AFL were observed in 20 out of 69 patients (29%) and only 2 of them showed symptoms. Initiation of an anticoagulation therapy was required in nine of them.

In late follow-up (after the first year) analysis, recurrent AF/AFL events in patients with a previous AF/AFL event during the early follow-up (in the first year) were excluded. Length of AF was >1 h in six patients and >24 h in two patients, but <1 h in all other patients. Episodes of ventricular tachycardia were found in 7.9% of patients within 2 years, with only one patient being symptomatic. Most of these were non-sustained VTs. Treatment changes (initiation of beta-blockers) were required in two of them, but no implantation of ICD devices was needed.

Further details on change in medical treatment are shown in Supplementary material online, *Table S5*.

### Clinical outcomes

The 2-year all-cause mortality was 19.3% (*n* = 17), with cardiovascular mortality accounting for less than half of fatal outcomes (*n* = 8). Number and type of arrhythmic events of patients who died are shown in Supplementary material online, *Table S6*. Two patients died from sudden death within the 2-year follow-up time after TAVR. Specific causes could not be established because their ILR could not be retrieved after death. Both cases occurred late during the follow-up period, one after 20 months and the other at 2 years, and both patients were known to have coronary artery disease. One of them had previously shown an episode of HAVB whereas the other had previously shown 12 episodes of HAVB, 17 severe bradycardia, and 1 NSVT. Treating physicians presumed such episodes were vagal. However, none of these conduction disturbances occurred within a few weeks before the sudden death episode. Unfortunately, no information on potential arrhythmic events was available for the last two days before their sudden death.

No ischaemic stroke events were reported. Two patients (2.3%) had major haemorrhagic strokes. One of them had AF at baseline and was on oral anticoagulation, whereas the other one did not show any AF episode at baseline and during the follow-up period.

## Discussion

In patients with NOP-LBBB after TAVR with an Evolut valve, late arrhythmic events occurred in about two-thirds of the patients and translated into a treatment change in about half of them. The PPM implantation was required in 12.5% of patients at 2 years. While tachyarrhythmic events, including AF/AFL, NSVT, AT, and VT, showed a gradual decrease in frequency throughout the 2-year period, bradyarrhythmic events showed a different temporal pattern. The rate of HAVB episodes was mostly early after the TAVR procedure and then remained stable over the 2-year follow-up, whereas episodes of severe bradycardia kept increasing after the first year.

Most studies assessing conduction disturbances after TAVR have used a 2-week continuous ambulatory ECG monitoring,^19^ except for the recent study by Massouillé *et al*. which assessed AV conduction disturbances by means of continuous ambulatory ECG monitoring in patients with NOP-LBBB up to 12 months after TAVR, and the study by Nozica *et al*. assessing new-onset arrhythmias within 12 months using an implantable cardiac monitoring,^11^ which provided a valuable overall insight on new-onset arrhythmias after TAVR. The current study reports specific data for the new-generation Evolut valves, a main device type with specific characteristics and design (difference in frame, radial force, position of the valve within the annulus, valve positioning manoeuvres, etc.) having a specific impact on the conduction system after TAVR implantation. Another recent study using novel digital technology showed that a wearable smartwatch was able to detect a large proportion of clinical events due to conduction abnormalities. However, the follow-up was still short (30 days).^12^ To the best of our knowledge, apart from the MARE study,^18^ only the Reveal study has assessed the incidence of arrhythmic events within 1 year, with AF being the most common arrhythmia in that cohort.^20^ Our study not only focuses specifically on patients with NOP-LBBB after TAVR, which are known to have a higher risk of conduction disturbances, but reported events at a follow-up of 2 years, showing that new-onset arrhythmic events still occur in a substantial number of patients beyond the first year after TAVR. Thus, selected patients might benefit from a long-term monitoring. Furthermore, our results provide now a better support to the generalizability of the findings, since our study included a number of centres in Canada, the USA, and Europe. This reflects current practice and management of these patients in diverse scenarios.

Nowadays, the need for a PPM due to procedure-induced conduction disturbances remains a major issue of TAVR, especially when expanding the indication to a lower risk population, since this has been shown to increase both mortality and morbidity/heart failure at midterm follow-up, including heart failure hospitalization.^21,22^ In our study, following an initial 4-week higher risk period, PPM implantations were homogenously distributed throughout the 2-year follow-up period. However, while the majority of patients receiving a PPM during the first year had HAVB, those receiving a PPM during the second year had other indications for implantation such as SND. These results are consistent with previous studies showing the most common cause of immediate (<1 month) post-procedural PPM implantation to be HAVB.^23^ However, in the long-term, other reasons such as SND become more important; therefore, electrocardiographic monitoring in the post-TAVR years may be necessary beyond the strict monitoring that is warranted immediately after the procedure.^4^

In the seminal clinical trial with the initial CoreValve device, the need for PPM at 1 year reached 22.3%.^24^ In subsequent clinical trials, use of CoreValve, Evolut R, or Evolut PRO in low-risk patients resulted in PPM implantation in 19.4% of patients at 1 year,^25^ whereas use of CoreValve or Evolut R in intermediate-risk patients in SURTAVI trial was associated to a 25.9% rate of PPM need at 30 days.^26^ Importantly, cusp-overlapping, a new promising valve implantation technique, has recently shown the lowest PPM rate to date (9.8%) in patients receiving the Evolut PRO/PRO+, in the Optimize PRO Study.^27^

Real-world and registry data on PPM rates after TAVR with successive generations of CoreValve/Evolut prostheses have been controversial. As a consequence of improvements in the stent frame of the valve, several comparative analyses have shown a substantially lower PPM rate with both Evolut R and Evolut PRO as compared to previous generation CoreValve devices.^6–8^ A systematic review suggested a 16.3–27.7% rate with CoreValve device vs. 14.7–26.7% rate with Evolut R.^28^ However, in spite of some observational comparative studies showing a significant reduction of PPM need with Evolut PRO as compared to Evolut R,^6,29^ other comparisons have only shown a non-significant trend to lower PPM rates^30,31^ or even a numerically higher PPM rate^7,9,10^ with Evolut PRO as compared to Evolut R.

In the general population, the reported annual rate of PPM implantation in patients with LBBB is 1–2%.^32^ In our study, the annual rate of PPM implantation was notably higher (6.2%). This difference suggests that TAVR patients with NOP-LBBB are at a higher risk of developing significant bradyarrhythmias and that the interaction of the prosthesis with the conduction system might play a role beyond the immediate effects of the implantation. For conduction disturbances appearing at longer term after TAVR, the pro-arrhythmic substrate that is inherent to the remodelled LV in elderly patients with AVS could play an additional role, besides the procedure itself. Current expansion of TAVR implantation to a younger population could help to elucidate the most probable aetiology of long-term conduction disturbances after TAVR.

Only 4 out of 11 patients receiving a PPM showed symptoms. This could have impacted on the percentage of implanted PPM in our study population, since indications were mainly based on monitoring findings. In line with these findings, the LOOP study showed that ILR screening led to a six-fold increase in bradyarrhythmia diagnoses and to a significant increase in pacemaker implantations compared to usual care, but no changes in the risk of syncope or sudden death were observed.^33^ These results show that arrhythmic events found in monitoring recordings do not always translate into clinical consequences. Future studies will need to focus on the time point at which action should be taken from a therapeutic point of view for these patients. Recently, measurement of HV intervals is gaining importance as a tool for risk stratification of post-TAVR conduction disorders in patients with persistent LBBB, as shown in a recent study by Massouillé *et al*.^34^ Although several studies have suggested different cut-off values (ranging from HV > 55 to 75) that could be useful to stratify this subgroup of patients,^34–36^ a definite cut-off value is yet to be established. In fact, some heterogeneity still exists in the management of conduction disorders at discharge after TAVR, but both ILR and HV interval measurement will need to be considered as stratification tools in this high-risk subgroup of patients.

The Evolut R valve consists of a self-expanding nitinol frame that conforms to the anatomy of the native annulus.^37^ Recently, Spaccarotella *et al*. documented a significant degree of acute expansion of nitinol prostheses immediately after deployment of the valve in patients treated with the Evolut R system.^38^ The impact of this further expansion in the long-term is still poorly understood with this new-generation device. However, previous studies with the old-generation CoreValve prosthesis suggest that the decrease in paravalvular leak (PVL) over time may be due to a continued expansion of the frame,^39^ which could translate into a potential increase in PPM rates over time (PVL/PPM trade-off). However, our study suggests that although some differences could appear with the new Evolut PRO, the low number of patients with this specific valve model included in our study prevents us from drawing any solid conclusions.

Some procedural characteristics such as depth of valve implantation are known to have an impact on conduction disturbances after TAVR. Jilaihawi *et al*. reported that both PPM and LBBB rates showed a substantial decline (from 9.7 to 3% for PPM and from 25.8 to 9% for LBBB) when implanting a self-expanding device (CoreValve/Evolut) above the membranous septum, as measured by multislice computed tomography.^40^ Thus, implantation depth appears to be a critical consideration in patients who are prone to develop conduction disturbances. Also, the use of a cusp-overlapping projection technique for implantation of the Evolut system has translated into a significant reduction in PPM likely due to the higher (more aortic) implantation of the transcatheter valve.^41^ Furthermore, some studies have shown septum length and thickness to be predictors for PPM.^42^

The two cases of CRT-D implantation in our study occurred within the second year of follow-up, due to a systolic dysfunction caused by ventricular dyssynchrony secondary to LBBB. This suggests that patients with a NOP-LBBB after TAVR could also benefit from continuous echocardiographic monitoring in order to identify those patients who could profit from a resynchronization device therapy or an implantable defibrillator (ICD) in the long term. The implantation of a conduction system pacing device (His or left bundle area pacing) could also improve heart failure symptoms. In addition, all patients in this trial received a standard RV apical pacing device which can contribute to left ventricular dysfunction or increase the risk of atrial fibrillation. Recent trials^43,44^ have shown the feasibility of conduction system pacing in the TAVR population which can prevent left ventricular dysfunction and also decrease the risk of atrial fibrillation and even ventricular arrhythmias.^45^ This new pacing approach may change the outcome in patients after TAVR who require a PPM.

Nearly one-third of the study population showed a new-onset AF/AFL event within the 2 years of follow-up; AF/AFL episodes occurred in one-fifth of the cohort in the first year and in one-tenth of the cohort in the second year after TAVR. Of note, most of these episodes were clinically silent, probably due to the short duration of the episodes. The 1-year incidence was higher than in previous trials like the US CoreValve High Risk trial (15.9%)^24^ or the Evolut Low-Risk trial (9.8%).^25^ On the other hand, a similar incidence was found in the NOTION (Nordic Aortic Valve Intervention) trial, probably due to the use of ILR to detect new-onset AF/AFL events.^46^ Thus, some events may go unnoticed if patients are asymptomatic or if only standard ECGs are recorded. Data on 2-year AF/AFL incidence after TAVR are scarce. Findings from the REVEAL AF study were consistent with our current results, with 29.3% of patients at high risk for AF showing an episode of AF/AFL within 18 months.^20^ Even though our data showed a reduction in frequency, a substantial rate of new-onset AF/AFL remains over time. The importance of detecting such events cannot be overemphasized, not only because of the need to prevent a potential risk of stroke but also because most of these patients will require an anticoagulation therapy that can also be associated with adverse effects. Notably, oral anticoagulation was only initiated in about half of patients with new-onset AF/AFL in our study. Many of the newly appeared AF episodes in our study had the characteristics of subclinical AF. The risk of stroke in these patients is slightly higher than in patients without subclinical AF but lower than in patients with clinically overt AF. Initiation of oral anticoagulation in such patients is still a matter of debate, but it can be considered in patients with subclinical AF > 24 h after careful clinical risk evaluation. In any case, the benefit of oral anticoagulation should outweigh the risk of bleeding.^47^

The GALILEO trial showed a higher risk for both thromboembolic and bleeding events in patients on oral anticoagulation (compared to those receiving an antiplatelet therapy) in patients without an indication for oral anticoagulation^48^; the selection of those patients with new episodes of AF/AFL requiring anticoagulants should therefore be made very carefully. Two very recent studies would further support this view. A pooled meta-analysis including the ARTESiA^49^ and NOAH-AFNET 6^50^ randomized trials in patients with short-duration atrial fibrillation (>6 min, <24 h) showed a reduced risk of stroke, but also an increased risk in major bleeding in patients with device-detected or subclinical AF.^51^ Based on these trials, shared decision-making with patients, discussing the risk of stroke or bleeding, and tailoring anticoagulation prescription to the high risk for stroke are favoured.^52,53^

Evidence on ventricular arrhythmias after TAVR is scarce, and its real impact on clinical outcomes after TAVR is still unknown. Studies on sudden cardiac death in patients with PPM after TAVR have shown ventricular tachycardia or ventricular fibrillation to be the cause of death in some cases.^54,55^ A previous study by Tempio *et al*. using continuous monitoring in patients who received either a CoreValve or a SapienXT prosthesis showed a very low rate of VT episodes (2%) and a significant reduction in ventricular arrhythmias at 1 month after TAVR.^56^ The cumulative events in our study were slightly higher (5.7% within the first year and 2.3% within the second year), although still decreasing over time. A potential explanation may be the improvement in systolic function resulting from the replacement of the stenotic valve itself, which could possibly reduce ventricular arrhythmic events. Despite the identification of non-sustained ventricular events in our study cohort, medical management was preferred (since patients had a left ventricular ejection fraction > 40%), and no ICD implantation was recommended.

### Study limitations

This study was based on a multicentre and international cohort of patients from both North America and Europe, and minor differences in the interpretation and management of arrhythmic events cannot be excluded. However, even though the initial diagnosis and therapy were performed in each individual site, all arrhythmic events were independently adjudicated in a single electrocardiography core lab. Other predictive factors for arrhythmic events could not be adequately assessed due to the non-controlled single-arm design and the limited sample size of the study. Although unlikely, similar arrhythmic findings cannot be excluded in patients without NOP-LBBB due to a missing control group. Even though all contributing centres participated in the early part of the study (up to 2016) with the expected number of patients, most centres stopped their participation in the later part of it due to budget constraints along with clinical restrictions during the COVID-19 pandemic. The potential impact of some anatomical factors such as implantation depth on conduction disturbances after TAVR could not be assessed. Since new pacing modalities such as conduction system pacing or leadless pacing were not used in this study, the impact of these technologies on arrhythmic burden will require further studies. Our results were obtained with the previous Evolut R valve and may not be generalized to the latest generation of the Evolut System (PRO/PRO+ and FX), potentially showing a different HAVB prevalence. However, the stent frame of the Evolut system is similar in all valve types, and the possibility of major differences in late arrhythmic events remains unlikely. Also, the number and the current follow-up length of patients having received these most recent devices are still limited, and it will take some time before a 2-year follow-up study to detect the late arrhythmic burden by means of an implantable recorder can be completed. In the meantime, our results are useful for management decision-taking in the large number of current patients having received the Evolut R device, and could be used as a reference to confirm or assess a potential change of such arrhythmic events after the implantation of newer devices. Finally, it is worth noting that our results are not generalizable to other types of valves such as balloon-expandable prostheses, due to their differences in design and implantation technique, which may imply a different frequency of conduction disturbances.

## Conclusions

In patients with NOP-LBBB after TAVR using a new-generation Evolut device, arrhythmic events were found in more than half of the study cohort within a 2-year follow-up period and translated into treatment changes in a significant proportion of cases. Although both tachyarrhythmic and bradyarrhythmic events seemed to decrease over time, PPM/ICD implantation still occurred during the second year of follow-up, which suggests a need for long-term monitoring in this subgroup of high-risk patients. As for events requiring PPM implantation, while the number of HAVB episodes remained relatively stable, SND became more important over time. The AF/AFL episodes represented a substantial proportion of all events, and this has important implications for the shared decision-making to start an anticoagulation regimen. The impact of new pacing modalities (leadless pacing or conduction system pacing) on arrhythmic burden in this population will require further studies. Finally, data on ventricular arrhythmias are still scarce in post-TAVR populations and should be investigated in future studies.

## Supplementary Material

euaf057_Supplementary_Data

## Data Availability

The data underlying this article will be shared on reasonable request to the corresponding author.
