# Supplementary material for: Late arrhythmic burden in patients with left bundle branch block after TAVR with the Evolut valve
Source: Europace. 2025 Mar 19;27(4):euaf057. doi: 10.1093/europace/euaf057 (PMC11983392; doi:10.1093/europace/euaf057)
Supplement: euaf057_Supplementary_Data [file euaf057_supplementary_data.docx]

**Supplementary Table 1. Arrhythmic events in early (≤ 1 year) and late follow-up (>1 year) (n=88)**

|  | **Overall period** | **First year** | **Second year** |
| --- | --- | --- | --- |
| **Global arrhythmic burden** | | | |
| Total number of new arrhythmic events | 411 | 307 | 104 |
| **Bradyarrhythmias ^a^** | | | |
| *Total number of events* | 101 | 45 | 56 |
| High-degree atrioventricular block | 35 | 22 | 13 |
| Severe bradycardia | 66 | 23 | 43 |
| **Tachyarrhythmias** | | | |
| *Total number of events* | 310 | 262 | 48 |
| Atrial arrhythmias | 303 | 257 | 46 |
| Atrial fibrillation/atrial flutter | 120 | 82 | 38 |
| Atrial tachycardia | 111 | 109 | 2 |
| Supraventricular tachycardia | 72 | 66 | 6 |
| Ventricular arrhythmias | 7 | 5 | 2 |
| Sustained ventricular tachycardia | 1 | 1 | 0 |
| Non-sustained ventricular tachycardia | 6 | 4 | 2 |
| **Atrial fibrillation/atrial flutter** | | | |
| Atrial fibrillation episodes per patient | 2 (1-8) | 2 (1-9) | 2 (1-5) |
| Duration of atrial fibrillation episodes per patient (min) ^b^ | 8 (0.13-50) | 40.5 (0.16-48.7) | 0.13 (0.12-30.4) |

Values are expressed as n or median (interquartile range).

^a^ Only patients without pacemaker or cardiac defibrillator implanted during the first year are considered in the denominator for the percentage. ^b^ Two patients with new chronic atrial fibrillation were excluded from the analysis.

**Supplementary Table 2.** Duration and symptoms of all severe bradycardia, non-sustained ventricular tachycardia and atrial fibrillation/atrial flutter events

|  |  |  |  |  |  |  |
| --- | --- | --- | --- | --- | --- | --- |
| **Severe bradycardia events** | | | | | | |
| **Patient** | **Date of procedure** | **Number of episodes** | **Date of event** | **Timing of event** | **Duration of event** | **Symptoms** |
| 250-005 | 24/09/2014 | 1 | 10/04/2015 | 198 | 00:00:03 | None |
| 250-005 | 24/09/2014 | 1 | 01/10/2015 | 372 | 00:00:03 | None |
| 250-005 | 24/09/2014 | 1 | 24/08/2015 | 334 | 00:00:03 | None |
| 250-005 | 24/09/2014 | 1 | 02/10/2015 | 373 | 00:00:04 | None |
| 250-005 | 24/09/2014 | 1 | 04/10/2015 | 375 | 00:00:03 | None |
| 250-012 | 22/04/2015 | 1 | 19/05/2016 | 393 | 0:00:03 | None |
| 250-012 | 22/04/2015 | 1 | 14/06/2016 | 419 | 0:00:03 | None |
| 250-012 | 22/04/2015 | 1 | 15/06/2016 | 420 | 0:00:03 | None |
| 250-012 | 22/04/2015 | 1 | 25/06/2016 | 430 | 0:00:03 | None |
| 250-012 | 22/04/2015 | 1 | 26/06/2016 | 431 | 0:00:03 | None |
| 250-012 | 22/04/2015 | 1 | 29/06/2016 | 434 | 0:00:03 | None |
| 250-012 | 22/04/2015 | 1 | 30/06/2016 | 435 | 0:00:04 | None |
| 250-012 | 22/04/2015 | 1 | 01/07/2016 | 436 | 0:00:03 | None |
| 250-012 | 22/04/2015 | 1 | 02/07/2016 | 437 | 0:00:03 | None |
| 250-012 | 22/04/2015 | 1 | 03/07/2016 | 438 | 0:00:03 | None |
| 250-012 | 22/04/2015 | 1 | 06/07/2016 | 441 | 0:00:03 | None |
| 250-012 | 22/04/2015 | 1 | 05/09/2016 | 502 | 0:00:03 | None |
| 250-012 | 22/04/2015 | 1 | 13/09/2016 | 510 | 0:00:03 | None |
| 250-012 | 22/04/2015 | 1 | 19/09/2016 | 516 | 0:00:03 | None |
| 250-012 | 22/04/2015 | 1 | 22/09/2016 | 519 | 0:00:03 | None |
| 250-012 | 22/04/2015 | 1 | 23/09/2016 | 520 | 0:00:03 | None |
| 250-012 | 22/04/2015 | 1 | 26/09/2016 | 523 | 0:00:03 | None |
| 250-30 | 24/05/2017 | 1 | 26/09/2017 | 125 | 0:00:03 | None |
| 250-30 | 24/05/2017 | 1 | 25/02/2018 | 277 | 0:00:05 | None |
| 250-51 | 15/03/2021 | 1 | 23/05/2021 | 69 | 0:00:03 | None |
| 250-51 | 15/03/2021 | 1 | 22/08/2021 | 160 | 0:00:05 | None |
| 250-51 | 15/03/2021 | 1 | 05/10/2021 | 204 | 0:00:05 | None |
| 250-51 | 15/03/2021 | 1 | 19/10/2021 | 218 | 0:00:04 | None |
| 252-4 | 26/03/2015 | 1 | 28/05/2015 | 63 | 0:00:03 | None |
| 254-7 | 07/07/2015 | 3 | 25/01/2017 | 568 | unknown | None |
| 255-020 | 23/02/2016 | 1 | 07/03/2016 | 13 | 00:00:04 | None |
| 255-024 | 27/04/2016 | 1 | 01/05/2016 | 4 | 00:00:06 | None |
| 255-026 | 18/01/2017 | 1 | 01/02/2017 | 14 | 00:00:03 | vasovagal episode |
| 317-5 | 10/11/2015 | 2 | 19/11/2015 | 9 | unknown | None |
| 317-6 | 06/01/2016 | 1 | 03/07/2017 | 544 | 00:00:05 | dizziness |
| 317-9 | 01/03/2016 | 1 | 29/03/2017 | 393 | 00:00:03 | None |
| 317-9 | 01/03/2016 | 1 | 28/05/2017 | 453 | 00:00:03 | None |
| 338-12 | 09/09/2016 | 1 | 20/09/2016 | 11 | 00:03:05 | dyspnea |
| 338-12 | 09/09/2016 | 1 | 20/09/2016 | 11 | 00:00:06 | dyspnea |
| 338-12 | 09/09/2016 | 1 | 20/09/2016 | 11 | 00:00:06 | dyspnea |
| 374-1 | 09/07/2015 | 5 | 30/01/2017 | 571 | 0:00:03 | dizziness |
| 374-6 | 07/07/2016 | 1 | 01/11/2017 | 482 | 0:00:06 | pre-syncope |
| 736-13 | 17/09/2019 | 1 | 17/09/2021 | 731 | 0:00:23 | pre-syncope |
| 753-11 | 09/05/2019 | 4 | 20/06/2019 | 42 | 0:00:34 | None |
| 753-12 | 27/09/2019 | 1 | 13/01/2020 | 108 | 0:00:31 | None |
| 753-12 | 27/09/2019 | 1 | 13/01/2020 | 108 | 0:00:13 | None |
| 753-12 | 27/09/2019 | 1 | 13/01/2020 | 108 | 0:00:13 | None |
| 753-12 | 27/09/2019 | 8 | 06/11/2020 | 406 | 0:00:10 | None |
| 753-24 | 05/10/2021 | 1 | 04/11/2022 | 395 | 0:00:10 | None |
|  |  |  |  |  |  |  |
|  |  |  |  |  |  |  |
| **Non-sustained ventricular tachycardia events** | | | | | | |
| **Patient** | **Date of procedure** | **Number of episodes** | **Date of event** | **Timing of event** | **Duration of event** | **Symptoms** |
| 250-010 | 13/02/2015 | 1 | 27/11/2015 | 287 | 00:00:07 | Dyspnea |
| 250-012 | 22/04/2015 | 1 | 12/02/2016 | 296 | 00:00:06 | None |
| 250-017 | 11/11/2015 | 1 | 14/08/2017 | 642 | 0:00:07 | None |
| 255-33 | 26/10/2018 | 1 | 29/07/2020 | 642 | 00:00:06 | None |
| 317-8 | 23/02/2016 | 1 | 08/03/2016 | 14 | unknown | None |
| 317-9 | 01/03/2016 | 1 | 02/06/2016 | 93 | unknown | None |

| **Atrial fibrillation/atrial flutter events** | | | | | | | |
| --- | --- | --- | --- | --- | --- | --- | --- |
| **Patient** | **Date of procedure** | **Number of episodes** | **Date of event** | **Timing of event** | **Type of event** | **Duration of event** | **Symptoms** |
| 250-017 | 11/11/2015 | 1 | 29/01/2017 | 445 | FA/flutter | 0:00:08 | None |
| 250-017 | 11/11/2015 | 1 | 28/02/2017 | 475 | FA/flutter | 0:00:06 | None |
| 250-017 | 11/11/2015 | 1 | 18/03/2017 | 493 | FA/flutter | 0:00:12 | None |
| 250-017 | 11/11/2015 | 1 | 23/03/2017 | 498 | FA/flutter | 0:00:07 | None |
| 250-017 | 11/11/2015 | 1 | 04/04/2017 | 510 | FA/flutter | 0:00:12 | None |
| 250-017 | 11/11/2015 | 1 | 22/04/2017 | 528 | FA/flutter | 0:00:09 | None |
| 250-017 | 11/11/2015 | 1 | 24/04/2017 | 530 | FA/flutter | 0:00:06 | None |
| 250-017 | 11/11/2015 | 1 | 01/06/2017 | 568 | FA/flutter | 0:00:07 | None |
| 250-017 | 11/11/2015 | 1 | 14/09/2017 | 673 | FA/flutter | 0:00:07 | None |
| 250-017 | 11/11/2015 | 1 | 16/11/2017 | 736 | FA/flutter | 0:00:08 | None |
| 250-017 | 11/11/2015 | 1 | 19/11/2017 | 739 | FA/flutter | 0:00:06 | None |
| 250-21 | 18/04/2016 | 1 | 03/05/2016 | 15 | FA | 02:00:00 | None |
| 250-21 | 18/04/2016 | 1 | 05/05/2016 | 17 | FA | 0:28:00 | None |
| 250-21 | 18/04/2016 | 1 | 23/05/2016 | 35 | FA | 0:00:06 | None |
| 250-21 | 18/04/2016 | 1 | 25/05/2016 | 37 | FA | 0:48:00 | None |
| 250-21 | 18/04/2016 | 1 | 26/05/2016 | 38 | FA | 0:50:00 | None |
| 250-21 | 18/04/2016 | 1 | 27/05/2016 | 39 | FA | 0:50:00 | None |
| 250-21 | 18/04/2016 | 1 | 13/06/2016 | 56 | FA | 1:16:00 | None |
| 250-21 | 18/04/2016 | 1 | 01/07/2016 | 74 | FA | 1:22:00 | None |
| 250-21 | 18/04/2016 | 1 | 03/07/2016 | 76 | FA | unknown | None |
| 250-21 | 18/04/2016 | 1 | 04/07/2016 | 77 | FA | 1:54:00 | None |
| 250-21 | 18/04/2016 | 1 | 06/07/2016 | 79 | FA | unknown | None |
| 250-21 | 18/04/2016 | 1 | 07/10/2016 | 172 | FA | 0:00:05 | None |
| 250-21 | 18/04/2016 | 1 | 24/11/2016 | 220 | FA | 0:00:05 | None |
| 250-21 | 18/04/2016 | 1 | 24/11/2016 | 220 | FA | 0:00:08 | None |
| 250-21 | 18/04/2016 | 1 | 14/03/2018 | 695 | FA | 0:00:08 | None |
| 252-10 | 29/12/2015 | 1 | 15/05/2017 | 503 | AF | 23:00:00 | None |
| 252-15 | 04/11/2016 | 1 | 25/11/2016 | 21 | AF | unknown | None |
| 254-12 | 20/06/2018 | 1 | 20/06/2019 | 365 | AF | 30 days | None |
| 255-28 | 26/07/2017 | 1 | 13/02/2018 | 202 | FA | 00:08:00 | None |
| 338-2 | 06/01/2016 | 1 | 04/10/2017 | 637 | FAP | 00:00:08 | None |
| 338-12 | 09/09/2016 | 1 | 20/09/2016 | 11 | AF | 00:04:00 | dyspnea |
| 338-12 | 09/09/2016 | 3 | 20/09/2016 | 11 | AF | 00:06:00 | dyspnea |
| 338-12 | 09/09/2016 | 1 | 18/12/2016 | 100 | AF | 00:24:00 | None |
| 338-12 | 09/09/2016 | 22 | 18/12/2016 | 100 | AF | 02:08:00 | None |
| 338-13 | 14/10/2016 | 1 | 29/11/2016 | 46 | AF | 00:22:00 | None |
| 338-13 | 14/10/2016 | 19 | 29/11/2016 | 46 | AF | 01:18:00 | None |
| 374-1 | 09/07/2015 | 1 | 15/07/2015 | 6 | AF | 0:00:10 | None |
| 374-1 | 09/07/2015 | 1 | 15/07/2015 | 6 | AF | 0:00:10 | None |
| 374-1 | 09/07/2015 | 1 | 26/10/2015 | 109 | AF | 0:00:15 | None |
| 374-1 | 09/07/2015 | 5 | 30/01/2017 | 571 | AF | 0:00:30 | None |
| 374-5 | 23/05/2016 | 1 | 01/01/2017 | 223 | Atrial flutter | 0:01:00 | None |
| 374-5 | 23/05/2016 | 1 | 01/01/2017 | 223 | Atrial flutter | 0:01:00 | None |
| 374-6 | 07/07/2016 | 2 | 01/11/2017 | 482 | FAP |  | None |
| 374-7 | 08/07/2016 | 1 | 15/07/2016 | 7 | AF | unknown | None |
| 374-7 | 08/07/2016 | 1 | 05/09/2016 | 59 | AF | unknown | None |
| 374-7 | 08/07/2016 | 1 | 13/02/2017 | 220 | AF | unknown | None |
| 374-7 | 08/07/2016 | 1 | 09/05/2017 | 305 | AF | unknown | None |
| 374-7 | 08/07/2016 | 1 | 04/09/2017 | 423 | AF | unknown | None |
| 374-7 | 08/07/2016 | 1 | 21/12/2017 | 531 | AF | unknown | None |
| 374-7 | 08/07/2016 | 1 | 12/03/2018 | 612 | AF | unknown | None |
| 374-7 | 08/07/2016 | 1 | 19/06/2018 | 711 | AF | unknown | None |
| 736-16 | 02/02/2020 | 5 | 13/01/2022 | 711 | AF | 1:00:00 | None |
| 736-16 | 02/02/2020 | 1 | 13/01/2022 | 711 | AF | 1:00:00 | None |
| 736-16 | 02/02/2020 | 1 | 13/01/2022 | 711 | AF | 1:00:00 | None |
| 736-16 | 02/02/2020 | 1 | 13/01/2022 | 711 | AF | 1:00:00 | None |
| 753-11 | 09/05/2019 | 1 | 13/01/2020 | 249 | AF | 0:08:00 | None |
| 753-11 | 09/05/2019 | 1 | 13/01/2020 | 249 | AF | 0:04:00 | None |
| 753-11 | 09/05/2019 | 1 | 09/02/2020 | 276 | AF | 7:30:00 | None |
| 753-11 | 09/05/2019 | 1 | 09/02/2020 | 276 | AF | 0:30:00 | None |
| 753-14 | 03/03/2020 | 2 | 26/03/2020 | 23 | AF | 0:46:03 | None |
| 753-16 | 07/05/2020 | 1 | 05/10/2021 | 516 | AF | 0:00:55 | None |
| 753-18 | 18/06/2020 | 1 | 03/06/2022 | 715 | AF | 0:00:02 | None |
| 753-19 | 16/07/2020 | 1 | 08/01/2021 | 176 | AF | 0:00:05 | None |
| 753-19 | 16/07/2020 | 1 | 25/01/2022 | 558 | AF | 0:00:07 | palpitations |
| 753-19 | 16/07/2020 | 1 | 25/01/2022 | 558 | AF | 0:00:05 | palpitations |
| 753-19 | 16/07/2020 | 1 | 25/01/2022 | 558 | AF | 0:00:05 | palpitations |
| 753-21 | 30/09/2020 | 1 | 21/11/2020 | 52 | AF | 99:99:99 | None |

**Supplementary Table 3**. Two-year global occurrence of first arrhythmic events (n=88)

|  | **First year** | **Second year** | **Overall period** |
| --- | --- | --- | --- |
| **Global arrhythmic burden** | | | |
| Patients with first arrhythmic event | 33 (37.5) | 13 (14.8) | 46 (52.3) |
| **Bradyarrhythmias** | | | |
| Patients with first bradyarrhythmic event | 16 (18.2) | 12 (13.6) | 28 (31.8) |
| Patients with high-degree atrioventricular block | 8 (9.1) | 6 (6.8) | 14 (15.9) |
| Pacemaker implantation^a^ | 6 (6.8) | 5 (5.7) | 11 (12.5) |
| **Atrial fibrillation/atrial flutter** | | | |
| Patients with new episodes of atrial fibrillation/atrial flutter^b^ | 13 (18.8) | 7 (10.1) | 20 (29.0) |
| **Ventricular tachycardia** | | | |
| Patients with new episodes of ventricular tachycardia | 5 (5.7) | 2 (2.3) | 7 (7.9) |
| Cardiac resynchronisation therapy defibrillator (CRT-D) ^c^ | 0 | 2 (2.3) | 2 (2.3) |

Values are expressed as n, n (%), n/N (%)

^a^ All pacemakers were implanted with an RV apical lead; no leadless or conduction system pacing was used ^b^ Only patients without prior atrial fibrillation are considered in the denominator for the percentage. ^c^ CRT-D were implanted based on heart failure, not on ventricular arrhythmias

**Supplementary Table 4**. Number of episodes, timing, duration and symptoms of patients developing complete atrioventricular block

| **Patient** | **Number of episodes** | **Timing of event (days)** | **Duration** | **Symptoms** | **Type of symptoms** |
| --- | --- | --- | --- | --- | --- |
| 1 | 1 | 6 | 00:00:02 | 1 | syncope |
| 2 | 2 | 9 | unknown | unknown |  |
| 3 | 1 | 30 | unknown | unknown |  |
| 4 | 1 | 43 | 00:00:03 | 0 |  |
| 5 | 4 | 48 | 0:00:12 | 0 |  |
| 6 | 1 | 182 | 0:00:04 | 0 |  |
| 7 | 1 | 195 | 0:00:03 | 1 | unknown |
| 8 | 1 | 301 | 00:00:03 | 0 |  |
|  | 1 | 302 | 00:00:04 | 0 |  |
|  | 1 | 305 | 00:00:03 | 0 |  |
|  | 1 | 308 | 00:00:03 | 0 |  |
|  | 1 | 309 | 00:00:04 | 0 |  |
|  | 1 | 312 | 00:00:03 | 0 |  |
|  | 1 | 314 | 00:00:04 | 0 |  |
|  | 1 | 316 | 00:00:03 | 0 |  |
|  | 1 | 318 | 00:00:03 | 0 |  |
|  | 1 | 323 | 00:00:03 | 0 |  |
|  | 1 | 327 | 00:00:03 | 0 |  |
|  | 1 | 337 | 00:00:03 | 0 |  |
| 9 | 1 | 382 | 00:00:03 | 0 |  |
| 10 | 1 | 386 | 00:00:04 | 0 |  |
| 11 | 1 | 393 | unknown | 0 |  |
| 12 | 1 | 395 | 0:00:04 | 0 |  |
|  | 1 | 395 | 0:00:04 | 0 |  |
| 13 | 1 | 460 | unknown | 1 |  |
| 14 | 1 | 759 | 0:00:04 | 1 | lipothymia |
|  | 1 | 759 | 0:00:02 | 1 | lipothymia |
|  | 1 | 759 | 0:00:02 | 1 | lipothymia |
|  | 1 | 759 | 0:00:02 | 1 | lipothymia |
|  | 1 | 759 | 0:00:02 | 1 | lipothymia |
|  | 1 | 759 | 0:00:02 | 1 | lipothymia |
|  | 1 | 759 | 0:00:02 | 1 | lipothymia |

**Supplemetary Table 5**. Details on medical treatment changes in patients with bradyarrhythmia, new atrial fibrillation/atrial flutter and ventricular tachycardia

| **Patients** | **Changes in medical treatment** | **Within the first year** | **Within the second year** |
| --- | --- | --- | --- |
| *Patients with bradyarrhythmia* | | | |
| 1 | Decreased dose of digoxin with subsequent increase after some months | 1 | 0 |
| 2 | Stop of betablocker | 1 | 0 |
| 3 | Stop of betablocker | 1 | 0 |
| 4 | Stop amiodarone | 1 | 0 |
| *Patients with new atrial fibrillation/atrial flutter* | | | |
| 1 | Start of anticoaogulation | 1 | 0 |
| 2 | Start of anticoaogulation | 0 | 1 |
| 3 | Start of anticoaogulation | 0 | 1 |
| 4 | Start of anticoaogulation | 0 | 1 |
| 5 | Start of anticoaogulation | 1 | 0 |
| 6 | Start of anticoaogulation | 1 | 0 |
| 7 | Start of anticoaogulation | 1 | 0 |
| 8 | Start of anticoaogulation | 1 | 0 |
| 9 | Start of anticoaogulation | 0 | 1 |
| *Patients with ventricular tachycardia* | | | |
| 1 | Start of atenolol | 1 | 0 |
| 2 | Adding of betablocker | 1 | 0 |

**Supplementary Table 6**. Cause of death, number and type of arrhythmic events in patients who died

| **Patient** | **Days of follow-up** | **Cause of death** | **Sudden cardiac death** | **Cardiovascular death** | **Number of new arrhytmic events** | **Type** |
| --- | --- | --- | --- | --- | --- | --- |
| 1 | 612 | Sudden cardiac death | 1 | 1 | 30 | 1NSVT/12AVB/17 pauses |
| 2 | 289 | Pneumonia | 0 | 0 | 0 |  |
| 3 | 521 | Multiorgan failure | 0 | 0 | 1 | AF |
| 4 | 30 | Non-cardiac | 0 | 0 | 0 |  |
| 5 | 182 | Unknown | Unknown | Unknown | 0 |  |
| 6 | 590 | Acute renal failure and heart failure | 0 | 1 | 0 |  |
| 7 | 740 | Complications of chronic heart failure | 0 | 1 | 0 |  |
| 8 | 37 | Pulmonary embolism | 0 | 1 | 0 |  |
| 9 | 34 | Congestive heart failure, pulmonary fibrosis and pneumonia | 0 | 1 | 1 | NSVT |
| 10 | 179 | Spontanous cerebral bleeding | 0 | 0 | 0 |  |
| 11 | 415 | Cardiac decompensation and acute renal failure | 0 | 1 | 0 |  |
| 12 | 743 | Sudden cardiac death | 1 | 1 | 1 | AVB |
| 13 | 35 | Hemorrhagic neurologic event | 0 | 0 | 0 |  |
| 14 | 16 | Subdural hematoma due to casual accident | 0 | 0 | 0 |  |
| 15 | 112 | Liver tumor | 0 | 0 | 0 |  |
| 16 | 350 | Unknown | Unknown | Unknown | 0 |  |
| 17 | 625 | Cardiac decompensation with acute renal failure | 0 | 1 | 0 |  |

AF: atrial fibrillation; AVB: atrioventricular block; NSVT: non-sustained ventricular tachycardia
